# Comparing High- Versus Low-Dose Entresto in Heart Failure Patients: A 2025 Meta-Analysis

**DOI:** 10.7759/cureus.90499

**Published:** 2025-08-19

**Authors:** Akshay Maharaj, Sajay Bidhesi, Sheneel Jaggernauth, Shyam R Ramoutar, Rajiv N Lutchmedial, Matthew A Maharaj, Aaron Lutchman, Amit Bhandari, Adam S Khan, Ramisa Ferdaus, Aparna Remanan, Mohammad M Husain, Sinead N Bhagwandeen, Victoria Bhagwandeen

**Affiliations:** 1 Internal Medicine, Port of Spain General Hospital, Port of Spain, TTO; 2 Internal Medicine, San Fernando General Hospital, San Fernando, TTO; 3 Medicine, The University of the West Indies, Champ Fleurs, TTO; 4 Medicine, Eric Williams Medical Sciences Complex, Champ Fleurs, TTO; 5 Clinical Sciences, University of the West Indies, St. Augustine, St. Augustine, TTO; 6 Medicine, The University of the West Indies at St. Augustine, St. Augustine, TTO; 7 Accident and Emergency, North Central Regional Health Authority, St. Joseph, TTO; 8 Anaesthesia and Intensive Care, Eric Williams Medical Sciences Complex, Champ Fleurs, TTO; 9 Internal Medicine, American University of the Caribbean School of Medicine, Cupecoy, SXM; 10 General Practice, University of the West Indies, St. Augustine, St. Augustine, TTO; 11 Internal Medicine, Bangladesh Medical College, Dhaka, BGD; 12 General Medicine, Sree Uthradom Thirunal Academy of Medical Sciences, Thiruvananthapuram, IND; 13 Internal Medicine, Gulf Coast Medical Center, Fort Myers, USA; 14 Internal Medicine, Howard University Hospital, Washington, USA; 15 Biology, College of Natural and Health Sciences, University of Tampa, Tampa, USA

**Keywords:** angiotensin receptor blocker (arb), angiotensin receptor-neprilysin inhibitor, cardiology research, entresto, heart failure with reduced ejection fraction, internal medicine-cardiology, sacubitril-valsartan

## Abstract

Entresto (sacubitril/valsartan) is widely used for the treatment of heart failure with reduced ejection fraction (HFrEF) due to its ability to enhance natriuretic peptide activity and inhibit the renin-angiotensin-aldosterone system (RAAS). However, its effects on various cardiovascular outcomes require further evaluation. Therefore, a meta-analysis of existing clinical studies was conducted to compare the effectiveness of high-dose vs. low-dose Entresto in treating chronic heart failure. A systematic search was performed using three databases: PubMed, ClinicalTrials, and Cochrane. The meta-analysis included nine randomized controlled trials comprising 4,011 patients with HFrEF. The inclusion criteria were: studies that evaluated patients (>18 years of age) with heart failure (these doses were generally divided into high (97/103 mg) and low (24/26 mg), with four studies using different dosing ranges relative to these categories); studies with relevant primary outcome; and studies written in English.

The primary outcomes include: heart failure hospitalization, all-cause mortality, left ventricular ejection fraction (LVEF), N-terminal prohormone of brain natriuretic peptide (NT-proBNP) levels, New York Heart Association (NYHA) functional class, and systolic blood pressure over a minimum follow-up period of three months.

We excluded studies with no quantitative outcome data, follow-up period (of at least three months), and dose-group comparisons. Additionally, studies were excluded if they were: case reports; meta-analyses; systematic reviews; conference abstracts; non-human studies or publications in any other language than English.

The risk of bias (RoB) was assessed using the Robvis tool and classified into low, moderate, serious, and critical risk of bias categories. The results of this assessment were illustrated via a traffic light plot.

A random effects statistical model was used in this study. Heterogeneity for LVEF was found to be substantial (I^2^ of 93.1%). Subgroup analysis found the source of this to be the dosing groups used in these studies, by excluding studies with less than or equal to 200 mg as their high-dose group. Heterogeneity for NT-proBNP was also substantial (I^2^ of 98%). Subgroup analysis could not localize a source of this heterogeneity, particularly when applied to dosing groups (high-dose groups with less than or equal to 200 mg of sacubitril/ valsartan).

The analysis revealed there was no statistically significant difference between LVEF improvement between low- and high-dose groups (1.95 (95% confidence interval (CI): -1.32 to 5.22); p=0.242). A meta-analysis of these studies showed a statistically significant reduction in NT-proBNP with Entresto. The pooled effect size was -667.24 (95% CI: -1312.69 to -21.8; p=0.04), indicating a significant decline in NT-proBNP levels in patients receiving a higher dose of Entresto. These findings suggest that while higher doses of Entresto do not significantly improve LVEF compared to lower doses, they are associated with a greater reduction in NT-proBNP levels, indicating improved cardiac stress and function. This supports the potential dose-dependent benefits of Entresto in managing heart failure with reduced ejection fraction. Further large-scale studies are warranted to explore the long-term impact of high-dose Entresto on clinical outcomes to optimize dosing strategies for HFrEF patients.

## Introduction and background

Heart failure is a clinical syndrome characterized by symptoms resulting from structural or functional cardiac abnormalities that impair ventricular filling or ejection. It may arise from diseases affecting the myocardium, pericardium, endocardium, heart valves, vessels, or metabolic dysfunctions. A key clinical measure in heart failure is the left ventricular ejection fraction (LVEF), which quantifies the percentage of blood ejected from the left ventricle during each contraction and helps classify heart failure into three subtypes: heart failure with reduced ejection fraction (HFrEF, LVEF<40%), heart failure with preserved ejection fraction (HFpEF, LVEF≥50%), and heart failure with midrange ejection fraction (HFmrEF, LVEF 41-49%) [1]. Another important biomarker is N-terminal pro-B-type natriuretic peptide (NT-proBNP), a blood marker that reflects cardiac stress and is used to assess heart failure severity and prognosis.

This study focuses on HFrEF and evaluates the comparative clinical impact of low-dose versus high-dose sacubitril/valsartan (marketed as Entresto), a medication that has become a cornerstone in its management. Sacubitril/valsartan is an angiotensin receptor-neprilysin inhibitor (ARNI) that combines neprilysin inhibition, which increases beneficial natriuretic peptides, and angiotensin II receptor blockade, thereby synergistically reducing cardiac remodeling and improving hemodynamics [2]. While the pharmacological benefits of sacubitril/valsartan are established, the optimal dosing strategy balancing maximum efficacy with tolerability remains unclear.

The PARADIGM-HF (Prospective Comparison of ARNI with ACEi (angiotensin-converting enzyme inhibitor) to Determine Impact on Global Mortality and Morbidity in Heart Failure) trial, the largest randomized controlled trial in HFrEF to date, enrolled approximately 8,400 patients and demonstrated that sacubitril/valsartan at the target dose of 97/103 mg twice daily reduced cardiovascular mortality by 20% and first heart failure hospitalization by 21% compared to enalapril [3,4]. NT-proBNP levels also decreased significantly with sacubitril/valsartan. However, PARADIGM-HF employed a run-in phase to exclude patients intolerant to therapy, and participants were closely monitored with high adherence. As a result, these findings may not fully generalize to routine clinical practice.

In real-world settings, tolerability and side effect profiles often limit the ability to escalate sacubitril/valsartan to its target dose. Observational data suggest that only 30%-50% of patients achieve the guideline-recommended dose of 97/103 mg twice daily [5,6]. The common adverse effects such as hypotension, hyperkalemia, and renal impairment are dose-dependent. For example, the incidence of hypotension ranges from 15% to 18% with high-dose therapy versus 5%-8% at lower doses, with similar trends observed for hyperkalemia and renal dysfunction [7-9]. These concerns lead many clinicians to maintain patients on submaximal doses in everyday practice.

While current guidelines recommend a stepwise titration to the highest tolerated dose to maximize neurohormonal blockade [10], the real-world discrepancy between recommended and achieved dosing highlights a significant clinical challenge. Prior studies investigating the dose-response relationship of sacubitril/valsartan have yielded conflicting results. Some real-world cohorts report improvements in LVEF and reductions in hospitalizations even at low or moderate doses, while others indicate superior outcomes only at target dosing [11,12]. These inconsistencies reveal a critical knowledge gap in optimizing sacubitril/valsartan therapy across diverse patient populations.

Given the widespread use of submaximal doses in practice and the ongoing uncertainty about their clinical effectiveness compared to target dosing, a comprehensive evaluation is needed. Therefore, this meta-analysis aims to systematically compare the effects of low-dose versus high-dose sacubitril/valsartan on clinical outcomes in HFrEF - including improvements in LVEF, heart failure hospitalizations, all-cause mortality, and adverse events. By synthesizing evidence from both randomized trials and real-world studies, this review seeks to clarify whether lower doses can offer comparable therapeutic benefits with improved tolerability, thereby informing individualized treatment strategies.

This analysis addresses a key clinical question: can lower, more tolerable doses of sacubitril/valsartan achieve similar outcomes to the recommended high-dose regimen? The results may support more nuanced, patient-centered dosing recommendations in the management of HFrEF.

## Review

This review was conducted in accordance with standardized methodological guidelines for systematic reviews and meta-analyses, adhering to the Preferred Reporting Items for Systematic Reviews and Meta-Analyses (PRISMA) framework. The risk of bias for each included study was evaluated using the Robvis tool (developed by Luke A. McGuinness of the University of Bristol), which provided visual assessments across core bias domains relevant to observational and cohort studies.

Search strategy

**Figure 1:**
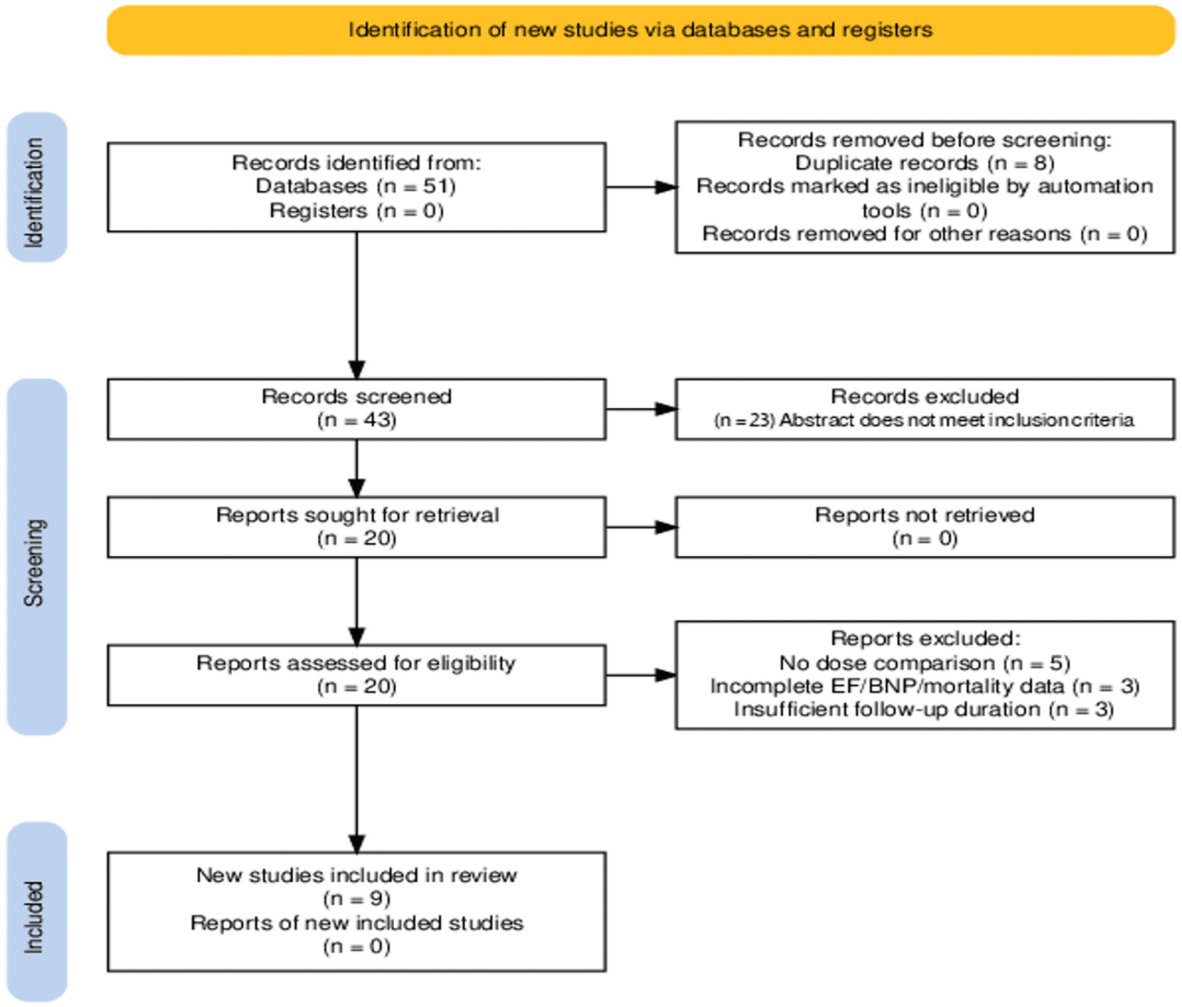
A stratified flow diagram of all studies identified, with screening in accordance with inclusion and exclusion criteria. EF: Ejection fraction; BNP: B-type natriuretic peptide.

A comprehensive literature search was carried out across major databases, including PubMed, Cochrane Library, and the Cochrane Central Register of Controlled Trials. Search terms included combinations of “heart failure” along with dose-related keywords such as “low dose,” “medium dose,” “high dose,” and related treatment-specific terminology. The search string applied for this process was as follows: ‘("heart failure" AND (sacubitril/valsartan OR entresto) AND (dose OR dosing OR underusing OR overdosing))’. Filters were applied to restrict the results to trials involving human participants, published in English, and conducted from inception to 2024, with no specific limits placed on study duration or geographical location (Figure 1).

Inclusion/exclusion criteria

Studies were included if they evaluated the effects of different dosing regimens - specifically low, medium, or high doses - of pharmacological treatments in adult patients (≥18 years) diagnosed with heart failure. Eligible studies were required to report at least one quantitative outcome relevant to cardiac function or clinical endpoints, such as ejection fraction (EF), B-type natriuretic peptide (BNP) levels, heart failure-related hospitalizations, or all-cause mortality. Only studies with an appropriate follow-up period of at least three months were used to assess and outcomes were considered. Acceptable study designs included prospective, retrospective, and observational cohort studies. Additionally, only full-text articles published in English between 2020 and 2024 were considered for inclusion.

Studies were excluded if they did not present quantitative outcome data, lacked follow-up or dose-group comparisons, or were published in languages other than English. Case reports, meta-analyses, systematic reviews, conference abstracts, and non-human studies were also excluded from the analysis. (Table 1).

**Table 1:** Summary of Included Studies Evaluating Dose-Dependent Effects of Sacubitril/Valsartan in HFrEF Patients. EF: Ejection fraction; SD: standard deviation; BNP: brain natriuretic peptide; HFrEF: heart failure reduced ejection fraction; M and F: males and females; ΔEF: mean difference ejection fraction; ΔBNP: mean difference BNP; SE: standard error; N=participants.

Author et al.	Year	Study Design	Location	N	Age	Gender	Follow-up Duration	High-Dose Mean Daily (mg)	Low-Dose Mean Daily (mg)	EF (Low Dose)	SD EF (Low Dose)	EF (High Dose)	SD EF (High Dose)	ΔEF	SE	BNP (Low Dose)	SD BNP (Low Dose)	BNP (High Dose)	SD BNP (High Dose)	ΔBNP	SE
Mohebi et al. [5]	2022	Prospective Cohort Study	USA	794	66.37±13.1	M and F	12 months	379	112	38.04	9.84	39.7	8.96	1.66	0.8238	527	52.2	308	39.1	-219	4.0007
Xie et al. [6]	2024	Retrospective Cohort Study	China	112	66.67	M and F	6 months	200	80	34	-	39	-	5	-	2200	-	1200	-	-1000	-
Martín-Garcia et al. [7]	2020	Retrospective Cohort Study	Spain	67	63±40	M and F	11 months	200	50	41.5	-	45	-	3.5	-	946	-	590	-	-356	-
Cheang et al. [8]	2022	Retrospective Cohort Study	China	983	57.3	M	6 months	200	50	35	11.017	37	10.887	2	1.083	2015	-	982	-	-1033	-
Monzo et al. [9]	2021	Retrospective Cohort Study	Italy	48	66.6±8.9	M and F	11 months	97/103	24/26	30.2	3.3	29.7	4.9	-0.5	1.5659	987	-	986	-	1	-
Corrado et al. [10]	2021	Prospective Observational Cohort Study	Italy	90	64±11	M and F	12 months	>75	<75	31	11	28	5	-3	1.7277	1680	1401	1613	1180	-500	272.7421
Park et al. [11]	2022	Retrospective Cohort Study	Korea	600	-	M and F	26 months	50	200	27.2	5.2	36.3	11.1	9.1	0.7463	-	-	-	-	-	-
Doi et al. [12]	2024	Retrospective Cohort Study	Japan	995	-	-	-	<200	400	37.9	15	39.4	14.4	1.5	1.0719	2984	1,233	1724	669.4	-1260	73.2359
Kido et al. [13]	2020	Retrospective Cohort Study	USA	322	-	M and F	3.5 years	97/103	24/26	-	-	-	-	-	-	-	-	-	-	-	-

Data analysis and interpretation

Three teams of two investigators each were tasked with screening studies based on the aforementioned inclusion and exclusion criteria, using Zotero (free to use tool originally developed by the Center for History and New Media at George Mason University and is now primarily developed by the Corporation for Digital Scholarship) to eliminate duplicates and using subgroup analysis to exclude studies with less than three months of follow-up. After narrowing down to nine studies, the effect size and standard error were input into JASP (version 0.19.0, JASP Team, 2024) to create a forest plot using measures of heterogeneity with a 95% confidence interval (CI). Chi values and degrees of freedom (df) were interpreted together, investigating the source of heterogeneity if Chi>df. Heterogeneity was measured by I^2^ with I^2^<50% and I^2^>50% needing further subgroup analysis to find the source of heterogeneity. Given the observed substantial heterogeneity in the analyses, indicated by high I² values, a random-effects model provides more conservative and generalizable estimates that account for variability across studies. Robvis was used to ascertain the level of bias among these studies.

Assessment risk of bias and quality of studies

The RoBvis risk of bias domains were used to analyze the bias categories of each study. Robvis-I classified each study into low, moderate, serious, and critical risk of bias. These results were obtained after each study was assessed with respect to domains, including the bias due to confounding, selection of participants, classification of intervention, deviations from intended intervention, missing data, measurement of outcomes, and in the selection of reported results.

**Figure 2:**
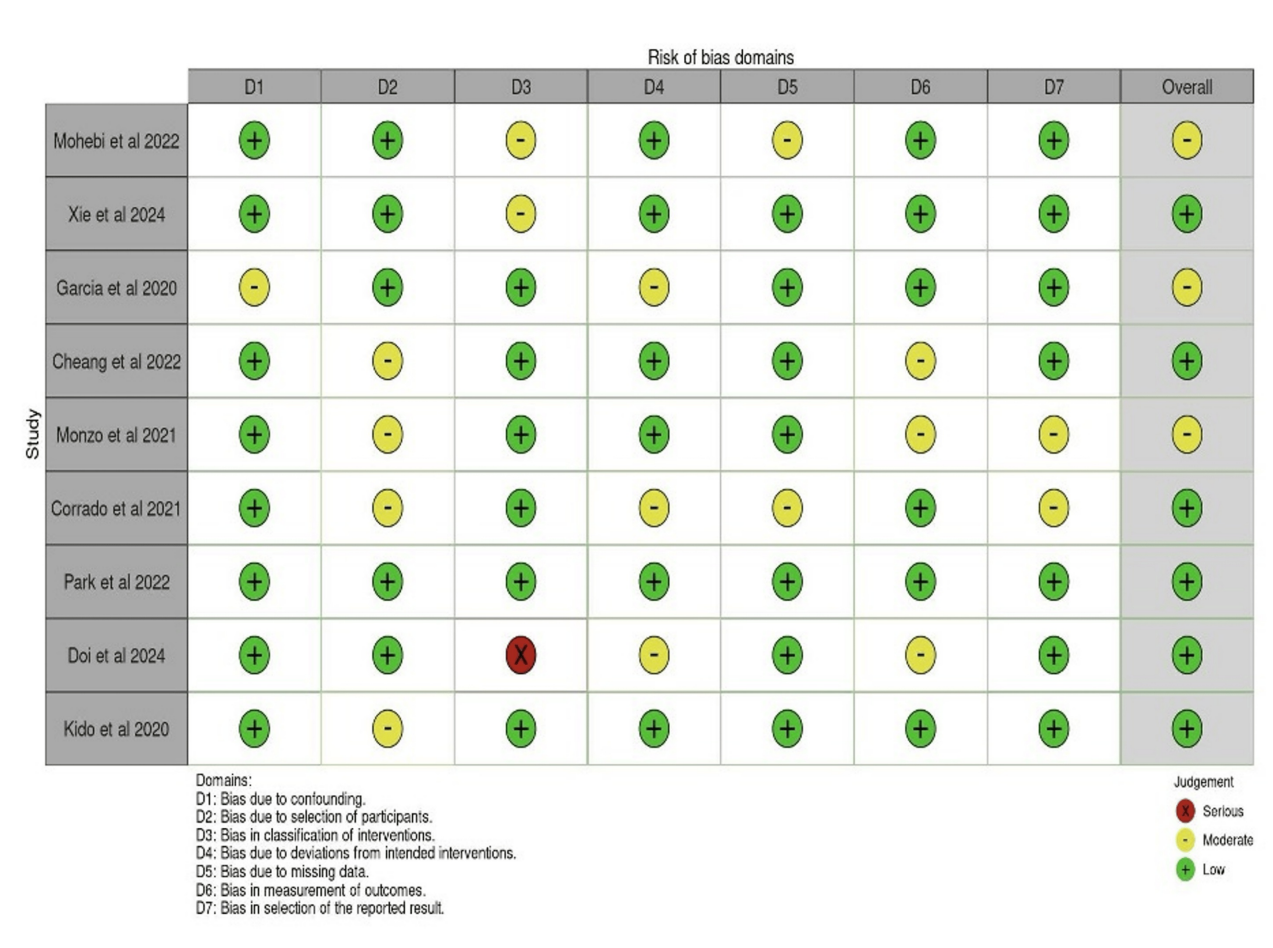
A Traffic light plot showing the different risk of bias for each study among each domain. Studies included: Mohebi et al. (2022) [5], Xie et al. (2024) [6], Garcia et al. (2020) [7], Cheang et al. (2022) [8], Monzo et al. (2021) [9], Corrado et al.(2022) [10], Park et al. (2022) [11], Doi et al. (2024) [12], and Kido et al. (2020) [13].

Quality of evidence extracted by two independent investigators with subsequent discrepancy analysis were employed, which was then settled by a third investigator via reasoning among both independent investigators. As per the traffic light plot (Figure 2), it is noted that the domain D2 (bias due to selection of participants) had the highest prevalence for risk of bias concerns. While these studies had some concern with respect to their selection of participants, it was not deemed to a serious concern. Therefore, the overall risk of bias was still low for most of the studies. In one study (Doi et al. (2024) [12]), there was a serious risk of bias in domain 3 due to ambiguous classifications of a singular intervention whereby low-dose Entresto was classified as <200 mg as opposed to more specific formulations [12]. Regardless, low-dose Entresto used in the study was still consistent with its outcomes and did not impact the results. This, coupled with low risk bias in the majority of the other domains, did not lead to overall serious bias in the study.

**Figure 3:**
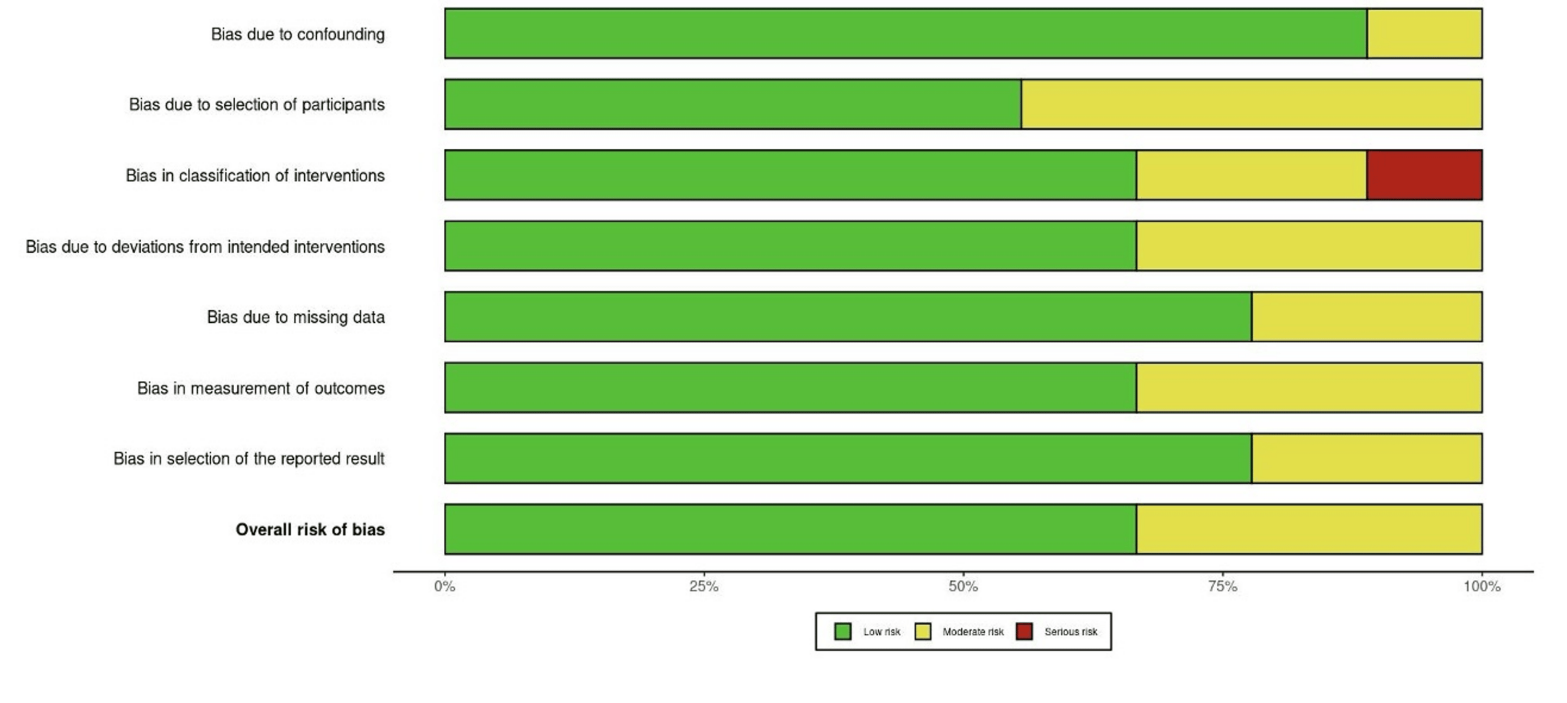
Bar graph comparing different domains and the cumulative risk of bias among the studies. Green=low bias; yellow=moderate bias; red=serious bias

As per the bar graph (Figure 3), bias due to confounding among the studies was the lowest. Meanwhile, the bias due to the selection of participants was the highest. Although there were some concerns for the risk of bias for some studies across the domains, it did not directly impact the overall risk of bias sufficiently. Hence, none of the studies were classified as having a serious overall risk of bias.

Results

We screened 792 abstracts published over the last nine years from 2016 to 2025. A total of 43 studies were retrieved for detailed evaluation, and nine studies were included. A total of 34 studies out of 43 were excluded, as the articles excluded did not meet the following inclusion criteria: (a) studies comparing low-dose Entresto (e.g., mean dose < 200 mg) with high-dose Entresto (e.g., mean dose ≥200 mg) in patients with heart failure, (b) follow-up duration ≥three months after the index day and (c) any of the following data: heart failure hospitalization, all-cause mortality, LVEF, NT-proBNP, NYHA functional class and systolic blood pressure (SBP). Subgroup analysis was done, as mentioned; however, due to the limited number of studies included, we were unable to fully investigate the sources of heterogeneity for some outcomes.

Primary analysis

LVEF Levels

**Figure 4:**
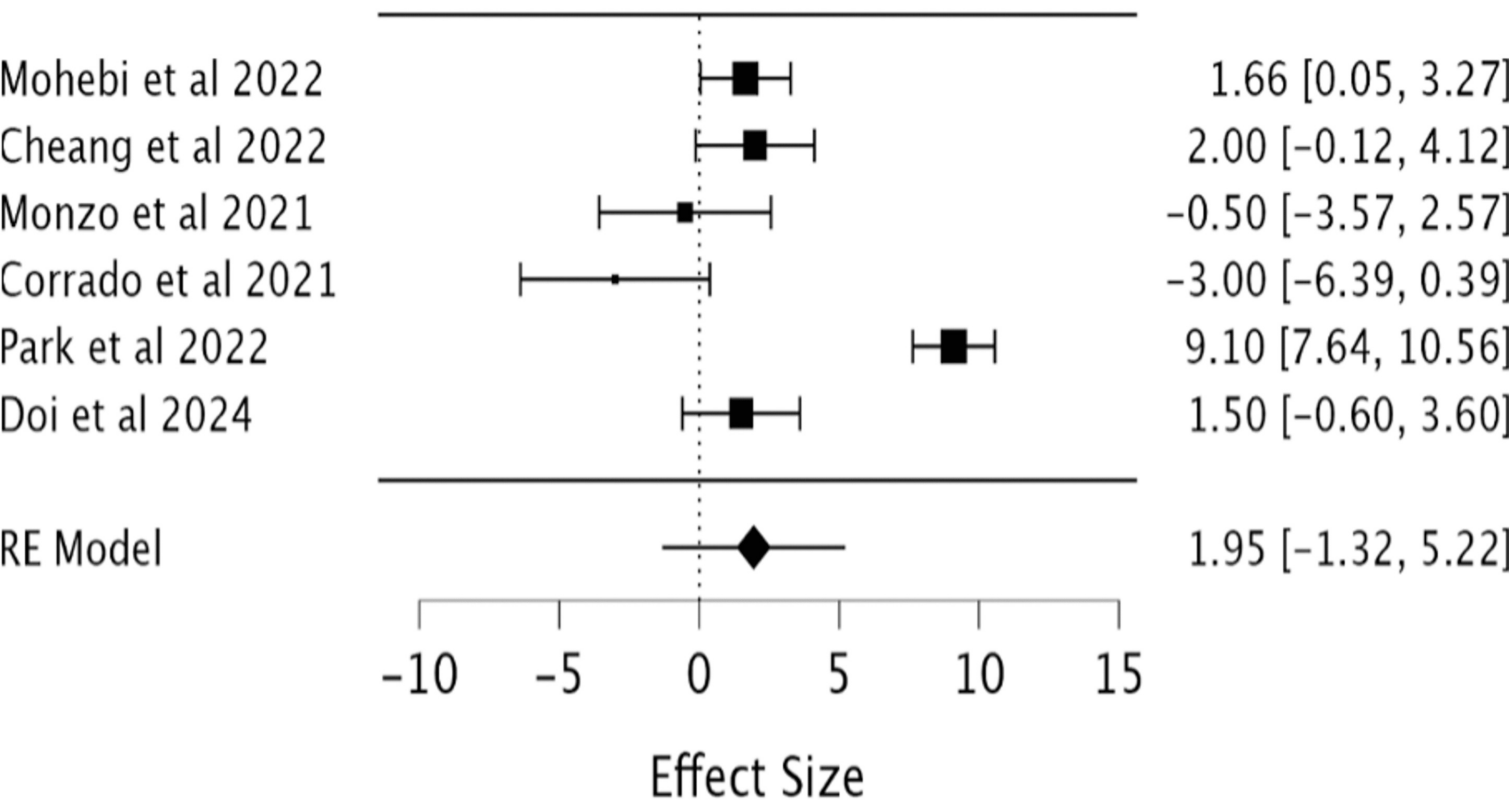
Forest plot showing the effect of Entresto on left ventricular ejection fraction. Studies included: Mohebi et al. (2022) [5], Cheang et al. (2022) [8], Monzo et al. (2021) [9], Corrado et al. (2022) [10], Park et al. (2022) [11], Doi et al. (2024) [12].

The nine studies included 4,011 patients with heart failure with reduced ejection fraction. All of the included studies were retrospective cohort studies, except for two, which were prospective cohort studies - Mohebi et al. (2022) [5] and Corrado et al. (2022) [10], as seen in Table 1. Four of the studies were performed in Asia, three in Europe and two in North America. Although a total of nine studies (4,011 patients) were included in our systematic review, only six were eligible for inclusion in the forest plot (Figure 4) for ejection fraction due to lack of data. The three excluded studies - Xie et al. (2024) [6], Garcia et al. (2020) [7], and Kido et al. (2020) [13] - did not report sufficient data on ejection fraction with some not providing mean LVEF values at baseline or follow-up, which prevented their inclusion in the pooled quantitative analysis.

There was no statistically significant difference between LVEF improvement and low-dose and high-dose groups. The confidence interval included the null value in our forest plot (Figure 4). The pooled effect size was 1.95 (95% CI: -1.32 to 5.22; Z=1.17, p=0.242).

*NT-proBNP Levels* 

**Figure 5:**
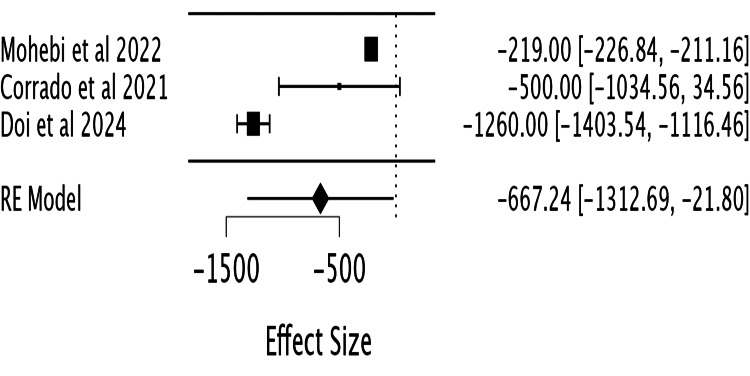
Forest plot evaluating the effect of Entresto on NT-proBNP Studies included: Mohebi et al. (2022) [5], Corrado et al. (2022) [10], Doi et al. (2024) [12].

Among the nine included studies, only three studies (Mohebi et al. (2022) [5], Corrado et al. (2022) [10], and Doi et al. (2024) [12]) reported sufficient data for inclusion in the forest plot evaluating the effect of Entresto on NT-proBNP levels (Figure 5). A meta-analysis of these studies showed a statistically significant reduction in NT-proBNP with Entresto. The pooled effect size was -667.24 (95% CI: -1312.69 to -21.8), indicating a significant decline in NT-proBNP levels in patients receiving a higher dose of Entresto. This result was statistically significant (Z=-2.02, p=0.04). However, substantial heterogeneity was observed (I²=98.3%, Chi²=202.4, df=2, p=0.001), suggesting variability across studies.

Heterogeneity and subgroup analyses

Subgroup Analysis for LVEF

Substantial heterogeneity was observed among the studies (I²=93.1%, df=5 (p=0.01), Chi²=87.3). Therefore, we proceeded to perform three subgroup analyses in order to identify the heterogeneity among the included studies. We first removed the Asian population from the subgroup; however, there was still no statistically significant result between high and low doses of Entresto. Secondly, we removed the subgroup that had follow-up for less than 12 months. For both of these subgroup analyses, we had maintained the conclusion from the original study.

In the third subgroup analysis, we removed studies in which the high dose was less than 200 mg (Monzo et al. (2021) [9], Corrado et al. (2022) [10], Park et al. (2022) [11], Kido et al. (2020) [13]). This gave us a new I² value of 0%, a df value of one, a pooled effect size of 1.78 (95%CI: 0.50, 3.07), a Chi² value of 0.06, and a Z value of 2.72. This showed that dosing was the source of heterogeneity. Therefore, there is a statistically significant rise in LVEF between high-dose and low-dose Entresto by 1.78. This dose-response relationship may reflect a biological threshold that must be met before the drug’s effect can be clinically appreciable. That is, the inhibition of neprilysin and the RAAS (renin-angiotensin-aldosterone system) may need a certain degree of its biochemical action to be exerted before any clinical differences are seen.

Subgroup Analysis for NT-proBNP 

For NT-proBNP, a subgroup analysis was conducted by excluding the study with a small sample size of 90 (Corrado et al. 2021 [10]), resulting in a pooled effect size of -736.36 (95% CI: -1757.08 to -283.22), with a high I² value of 98.3% (Chi²=201.4, df=1, p=0.167). This indicates that the exclusion of the small sample size study did not substantially alter the effect of Entresto on NT-proBNP levels, though there is considerable heterogeneity.

Lastly, we attempted to perform a subgroup analysis excluding studies with a high-dose Entresto dosage of less than 200 mg (Monzo et al. (2021) [9], Corrado et al. (2022) [10], Park et al. (2022) [11], Doi et al. (2024) [12], Kido et al. (2020) [13]). However, due to the insufficient number of studies remaining (fewer than two), this analysis could not be performed, limiting further investigation into the impact of dose on NT-proBNP levels.

Publication bias 

LVEF

**Figure 6:**
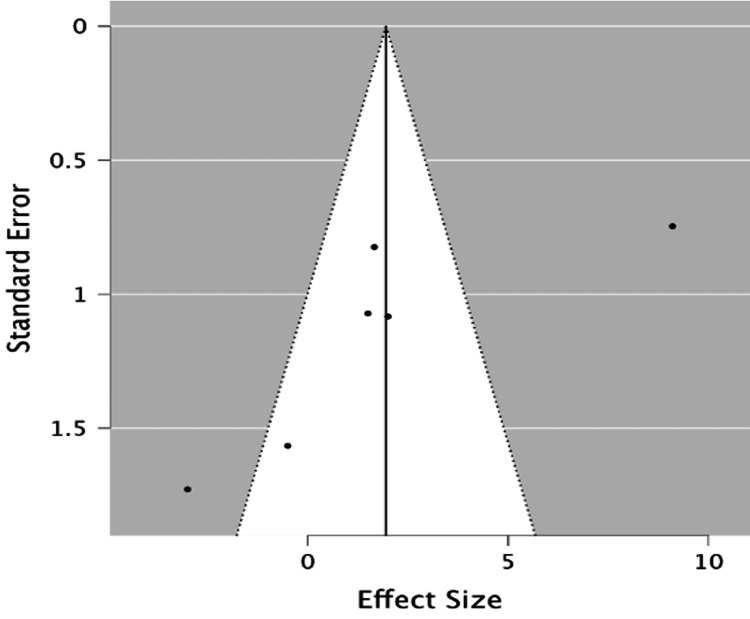
A funnel plot for left ventricular ejection fraction.

We created a funnel plot for ejection fraction (Figure 6), in which we found an even distribution. The p-value for Egger’s test was 0.008. That indicates that there is publication bias for the studies used.

NT-pro BNP

**Figure 7:**
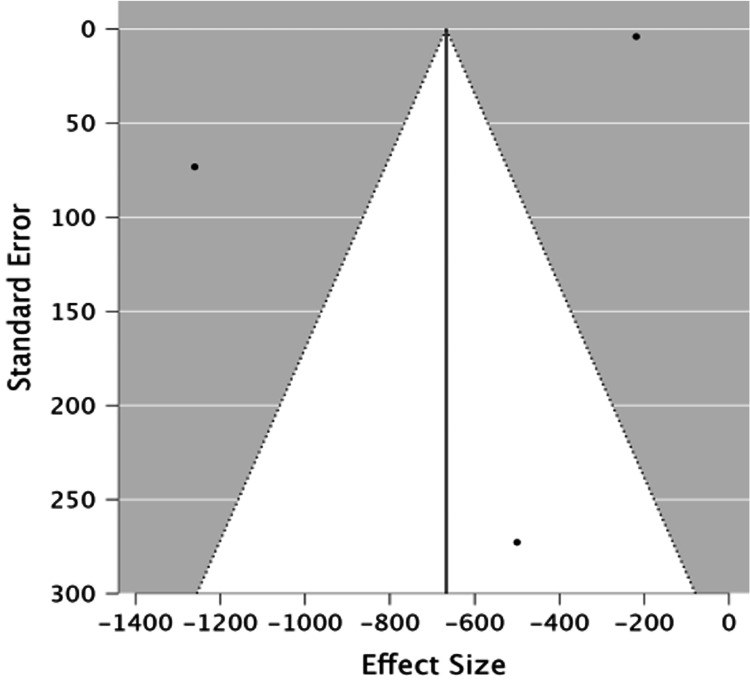
A funnel plot showing NT-proBNP

We created a funnel plot for NT-proBNP (Figure 7), which showed a wide and even distribution of studies. The p-value for Egger’s test was 0.99, suggesting no substantial evidence of publication bias in the studies included in the BNP analysis.

Discussion

In this meta-analysis of seven retrospective and two prospective studies comprising 4,011 patients, we assessed the efficacy of low-dose versus high-dose Entresto in patients, focusing particularly on LVEF and NT-proBNP levels. Our meta-analysis found no statistically significant difference in LVEF improvement between the low-dose and high-dose groups (pooled effect size 1.95; 95% CI: -1.32 to 5.22; Z=1.17; p=0.242). However, there was a statistically significant reduction in NT-proBNP with a higher dose of Entresto (pooled effect size -667.24; 95% CI: -1312.69 to -21.8; Z=-2.02; p=0.04). The substantial heterogeneity observed (I²=98.3%) suggests considerable variability across studies.

HFrEF is characterized by impaired systolic function and remains a major cause of morbidity and mortality. Optimizing medical therapy in HFrEF aims to reverse adverse remodeling, improve LVEF, reduce hospitalizations, and lower mortality. The PARADIGM-HF trial demonstrated that sacubitril/valsartan at the target dose (97/103 mg twice daily) significantly reduced cardiovascular death and heart failure hospitalization compared to enalapril [14]. Also, a comparative study by Tan et al. found that sacubitril-valsartan was associated with lower risks of death and hospitalization compared with angiotensin-converting enzyme (ACE)/angiotensin receptor blocker (ARB) in a heterogeneous cohort of patients with systolic HF [15]. Similarly, the PROVE-HF study showed that reductions in NT-proBNP correlated strongly with improvements in LVEF and cardiac structure [16]. The TITRATION (Comparison of Two Treatment Initiation Regimens in Heart Failure Patients With Reduced Ejection Fraction) trial further confirmed that careful dose up-titration maximizes benefits while balancing tolerability [17,18]. The PIONEER‐HF (Comparison of Sacubitril-Valsartan versus Enalapril on Effect on NT-proBNP in Patients Stabilized From an Acute Heart Failure Episode) trial also confirmed that initiating sacubitril/valsartan in hospitalized patients, followed by careful dose titration, led to favorable outcomes with an acceptable safety profile [19].

Our findings, however, reveal a notable discordance between biomarker and functional outcomes. While NT-proBNP reduction was significant with higher doses, LVEF improvement was not significantly different between dose groups overall. This discrepancy may reflect several factors: NT-proBNP is a sensitive marker of myocardial stress and may respond earlier or more robustly to therapy than structural remodeling captured by LVEF, which can be influenced by measurement variability, timing, and patient heterogeneity. Additionally, the high heterogeneity among included studies suggests variable patient populations and methodologies that could dilute the observed effects. An illustrative example is the meta-analysis by Chen et al., which demonstrated that, compared with low-dose sacubitril/valsartan, high-dose therapy was associated with a markedly lower risk of heart failure hospitalization and all-cause mortality. In contrast, no significant differences were observed between low- and high-dose groups in terms of improvements in NYHA functional class, changes in left ventricular ejection fraction, alterations in NT-proBNP levels, or changes in systolic blood pressure [20]. Potential explanations for these findings include heterogeneity in the operational definitions of “low” and “high” dose across studies and measurement limitations for NYHA classification, which is inherently subjective and susceptible to inter-observer variability and the variability of systolic blood pressure, which can be influenced by hydration status, concomitant medications, and diurnal variation, thereby obscuring group-level differences. Furthermore, NT-proBNP levels are modulated by factors beyond left ventricular unloading, such as atrial fibrillation, renal function, and obesity, which may attenuate the apparent dose-response relationship in pooled analyses.

Importantly, our subgroup analysis demonstrated that when low-dose studies were excluded, LVEF improvement with high-dose Entresto became statistically significant. This finding aligns with prior evidence supporting up-titration to target doses for maximal remodeling benefits [14,17,18]. It underscores the potential limitation of low-dose therapy in achieving optimal LVEF gains, although low doses may still confer some biomarker and symptomatic improvements.

While we discuss vulnerable populations such as elderly patients or those with renal impairment, it is important to note that our meta-analysis did not stratify or specifically analyze these subgroups due to limited reporting in the included studies. Thus, discussions regarding low-dose strategies in these groups remain largely theoretical based on clinical experience and prior observational data [13]. The clinical relevance of low-dose Entresto in real-world high-risk populations is an area warranting further targeted research.

Regarding reasons for not up-titrating patients on low-dose Entresto, hypotension was significantly more common in patients who did not reach high-dose therapy, while rates of hyperkalemia, severe renal events, and angioedema were similar across dose groups. Moreover, attaining high-dose therapy was positively associated with higher baseline systolic blood pressure, better geriatric nutritional risk index (GNRI), lower NYHA class, preserved renal function, and male sex, suggesting comorbidities and low baseline blood pressure were major barriers to up-titration.

In summary, although low-dose ARNI therapy may offer practical benefits in tolerability, our meta-analysis indicates that it may not provide significant LVEF improvement comparable to higher doses. Clinicians should weigh the modest efficacy of low-dose regimens against the risks and feasibility of up-titration, especially in frail or comorbid patients. Our findings emphasize the need for individualized treatment strategies supported by further research focusing on vulnerable subpopulations and real-world effectiveness.

Clinical relevance

LVEF is a critical measure of the heart’s pumping efficiency, reflecting the percentage of blood ejected by the left ventricle with each contraction. Clinically, a low LVEF (typically <40%) indicates impaired heart function and is associated with increased mortality and morbidity. While improving LVEF through medical interventions such as sacubitril/valsartan (Entresto) is associated with symptomatic relief and better long-term outcomes, our meta-analysis did not find a statistically significant difference in LVEF improvement between low- and high-dose groups overall. This suggests that functional improvements may be dose-dependent or require longer follow-up to manifest clearly.

NT-proBNP is a biomarker released in response to myocardial stress and volume overload in heart failure. Elevated NT-proBNP levels correlate with worse clinical outcomes, and reductions in NT-proBNP have been associated with improved prognosis. Our meta-analysis showed a statistically significant reduction in NT-proBNP with higher doses of sacubitril/valsartan, supporting a beneficial biological effect at the biomarker level.

Taken together, these findings indicate that while higher doses of sacubitril/valsartan may offer greater biomarker improvement, the impact on functional cardiac recovery as measured by LVEF remains less certain. Clinical decision-making should therefore carefully consider patient tolerability and risk factors when pursuing dose optimization. Future research with larger, dose-stratified trials is needed to clarify how dosing strategies can be best individualized to maximize both biomarker and functional benefits. This meta-analysis provides an important context for providers weighing the potential advantages and limitations of sacubitril/valsartan dosing in heart failure management. These biomarkers act as surrogates for the systolic function of the heart and cardiac wall stress, respectively. However, they do not offer a direct view of the heart’s status; these measures are better used as additional evidence to corroborate with clinical and other investigative findings to determine patient-centered outcomes, disease progression, hospitalization, and mortality rates.

Implications for future research

Entresto (sacubitril/valsartan) is indicated for the treatment of HFrEF and is recommended for patients who remain symptomatic despite optimal ACE inhibitor or ARB therapy or who are intolerant to ACE inhibitors. Based on our meta-analysis findings, future research should prioritize large-scale, long-term studies that compare clearly defined dosing regimens, particularly focusing on low-dose (24/26 mg), intermediate-dose (49/51 mg), and high-dose (97/103 mg) sacubitril/valsartan. This approach could clarify dose-response relationships and optimize individualized treatment strategies.

Although our study did not assess NYHA functional class, future investigations should include this parameter due to its widespread use in clinical practice for stratifying heart failure severity and guiding therapy. Evaluating how Entresto’s efficacy and tolerability vary across NYHA classes could help tailor treatment recommendations to patient symptom burden and functional status.

Additionally, research should explore subgroup outcomes based on age, comorbidities, hospitalization frequency, and mortality to address current gaps in understanding Entresto’s benefits in diverse real-world populations. This would strengthen evidence-based dosing guidelines and enhance personalized care.

Limitations

The limitations of this meta-analysis were either methodological or clinical. The methodological limitations include the study size; due to the extensive exclusion criteria, the number of studies included in this meta-analysis was relatively small, which limited the results of the study and its ability to generate useful conclusions. Heterogeneity of the included studies was also a limitation; within this meta-analysis, there may be variability within the studies analyzed regarding patient demographics, comorbidities, and study designs, which may affect the interpretation and applicability of the findings across different dosage groups. Despite multiple inclusion criteria, individual studies within this meta-analysis may still carry biases (e.g., selection bias, performance bias) that could affect the reliability of the combined results. Subgroup analysis of high-dose Entresto groups resulted in greater significance. From this, a potential limitation would be the inclusion of studies focused on low-dose Entresto or whose high-dose group was a mean daily dose of less than 200 mg (inconsistent dosing definitions), potentially restricting the comprehensive evaluation of high-dose Entresto’s full potential. Furthermore, the method used in a meta-analysis, while valuable for synthesizing data from multiple studies, is dependent on the quality and consistency of the included studies and any biases or methodological flaws in those studies can skew the results. Furthermore, meta-analyses also cannot account for factors not measured in the original studies, such as unmeasured confounders, and may not provide insights into individual patient responses. Additionally, they can be limited by publication bias, where studies with negative or inconclusive results are less likely to be published and included. Finally, despite numerous studies being conducted on low-dose versus high-dose Entresto groups, they assess a wide variety of patient outcomes not included in our study’s inclusion criteria, limiting our ability to comprehensively compare these two groups.

The clinical limitations include the use of additional treatment in the placebo group or the addition of the tolerable dose of Entresto as part of a polydrug regimen, which could influence comparisons between the high-dose and placebo groups. It should also be noted that Entresto at any dose would not be used as monotherapy, so there may be study heterogeneity depending on exact pharmacological combinations and dosages used in the control group. Furthermore, a meta-analysis of ARNI dosing also supports that while a dose-response relationship exists, the risk-benefit profile in patients with low baseline blood pressure or other risk factors (such as renal dysfunction or hyperkalemia) may favor a more conservative titration strategy (Senni et al., 2016 [17]). Addressing these limitations in future research could strengthen the validity and applicability of our findings.

Final thoughts

This meta-analysis consolidates the current evidence on the impact of low-dose versus high-dose Entresto in patients with HFrEF and its effect on LVEF and NT-proBNP. The results show a statistically significant decrease in NT-proBNP with high-dose Entresto as compared to low-dose Entresto. An increase in LVEF levels was noted; however, no statistically significant benefit was found from higher doses of Entresto as compared to lower doses. In summary, these findings indicate a positive effect specifically on the NT-proBNP biomarker with high-dose Entresto. However, despite a positive trend (towards improvement) noted for the LVEF, it remains statistically insignificant. Furthermore, though these biomarkers are used as surrogates for clinical outcomes, the broader impact on long-term, patient-centered, clinical outcomes remains uncertain based on current data. Further research comparing various doses of Entresto and monitoring a variety of clinical outcomes is essential to better define its role in the management of cardiovascular disease and optimize its therapeutic potential.

## Conclusions

This meta-analysis suggests that higher doses of Entresto significantly reduces NT-proBNP, indicating a positive impact on heart failure biomarkers. However, despite an observed improvement in LVEF, the difference between doses (high versus low) was not statistically significant. Several factors limit the degree of certainty of these findings, such as the robust exclusion criteria as well as the variable high-dose category used in the residual studies that were compared to their low-dose studies. However, these findings indicate a general tolerance to high doses of Entresto in multiple study populations, as evidenced by the decreased incidence of side effects that warranted cessation of therapy when compared to mainstays of the treatment algorithm such as enalapril. Further research comparing different dosing strategies and assessing a broader range of clinical outcomes is essential to optimize its therapeutic potential in cardiovascular disease management.
